# An automated structured education intervention based on a smartphone app in Chinese patients with type 1 diabetes: a protocol for a single-blinded randomized controlled trial

**DOI:** 10.1186/s13063-020-04835-9

**Published:** 2020-11-23

**Authors:** Fansu Huang, Xinyin Wu, Yuting Xie, Fang Liu, Juan Li, Xia Li, Zhiguang Zhou

**Affiliations:** 1 National Clinical Research Center for Metabolic Diseases and Department of Nutrition, The Second Xiangya Hospital, Central South University, Changsha, 410011 China; 2 National Clinical Research Center for Metabolic Diseases, Key Laboratory of Diabetes Immunology, Ministry of Education, and Department of Metabolism and Endocrinology, The Second Xiangya Hospital, Central South University, Changsha, 410011 Hunan China; 3 Xiangya School of Public Health, Central South University, Changsha, 410011 China; 4 Clinic Nursing Teaching and Research Section, The Second Xiangya Hospital, Central South University, Changsha, 410011 China

**Keywords:** Automated structured education, Type 1 diabetes, Randomized controlled trial, Intervention, Artificial intelligence, Smartphone application (app)

## Abstract

**Background:**

Although evidence had demonstrated the effectiveness of smartphone apps in diabetes care, the majority of apps had been developed for type 2 diabetes mellitus (T2DM) patients and targeted at populations outside of China. The effects of applying a smartphone app with structured education on glycemic control in type 1 diabetes mellitus (T1DM) are unclear. A digital, culturally tailored structured education program was developed in a smartphone app (Yi tang yun qiao) to provide an automated, individualized education program aimed at improving self-management skills in patients with T1DM in China. This trial aims to investigate the effectiveness of this smartphone app among Chinese T1DM patients.

**Methods and analysis:**

This single-blinded, 24-week, parallel-group randomized controlled trial of a smartphone app versus routine care will be conducted in Changsha, China. We plan to recruit 138 patients with T1DM who will be randomly allocated into the intervention group (automated, individualized education through an app) or routine care group. The intervention will last for 24 weeks. The primary outcome will be the change in glycated hemoglobin (HbA1c) from baseline to week 24. The secondary outcomes will include time in range, fasting blood glucose, levels of serum triglycerides and cholesterol, blood pressure, body mass index, quality of life, diabetes self-care activities, diabetes self-efficacy, depression, anxiety, and patient satisfaction. Adverse events will be formally documented. Data analysis will be conducted using the intention-to-treat principle with appropriate univariate and multivariate methods. Missing data will be imputed with a multiple imputation method under the “missing at random” assumption.

**Discussion:**

This trial will investigate the effectiveness of an app-based automated structured education intervention for Chinese patients with T1DM. If the intervention is effective, this study will provide a strategy that satisfies the need for effective lifelong diabetes care to reduce the disease burden and related complications resulting from T1DM.

**Trial registration:**

ClinicalTrials.gov NCT04016987. Registered on 29 October 2019.

## Data set

**Table:** 

Data category	Information
Primary registry and trial identifying number	ClinicalTrials.gov: NCT04016987
Date of registration in primary registry	29 October 2019
Secondary identifying numbers	N/A
Source(s) of monetary or material support	National Key R&D Program of China
Primary sponsor	The Second Xiangya Hospital
Secondary sponsor(s)	N/A
Contact for public queries	XL, MD, phone: +86 0731-85292154, email: lixia@csu.edu.cn
Contact for scientific queries	XL, MD, The Second Xiangya Hospital, Central South University, Changsha, China
Public title	An automated structured education intervention based on a smartphone app in Chinese patients with type 1 diabetes: protocol for a randomized controlled trial
Scientific title	An automated structured education intervention based on a smartphone app in Chinese patients with type 1 diabetes: a protocol for a single-blinded randomized controlled trial
Countries of recruitment	China
Health condition(s) or problem(s) studied	Type 1 diabetes mellitus
Intervention(s)	1. Automated, individualized education group: automated, individualized education based on through a smartphone app; 2. Routine care group: education through health-care professionals in the outpatient department
Key inclusion and exclusion criteria	Inclusion criteria: individuals with a T1DM duration of over 6 months who own a smartphone and are capable of using a smartphone app and WeChat, treated with multiple daily injections or an insulin pump, aged 18–50 years, and HbA1c > 7%. Exclusion criteria: being pregnant, having mental disorders, having other conditions or chronic complications related to T1DM, and already using a smartphone app to manage diabetes.
Study type	Interventional; Allocation: randomized; Intervention model: paralleled assignment Masking: single-blind (outcomes assessor) Primary purpose: treatment; Phase: N/A
Date of first enrolment	November 2020
Target sample size	138
Recruitment status	Recruiting
Primary outcome(s)	Hemoglobin A1c level (time frame: 24 weeks)
Key secondary outcomes	Physiological parameters, psychosocial outcomes, and psychological outcomes (time frame: 24 weeks)

## Strengths and limitations of this study

➢ The smartphone app evaluated in this trial was developed in a theoretical- and evidence-based manner and contains a digital culturally tailored structured education program specifically targeted at T1DM patients in the Chinese population.

➢ In combination with artificial intelligence technology, automated, individualized education was offered to a group of patients to explore whether tailoring education materials provided to patients might enhance the effects of the smartphone app.

➢ Treatment effects will be measured with a wide spectrum of outcomes, including physiological parameters, psychosocial outcomes, and psychological outcomes, to capture the complex nature of type 1 diabetes.

➢ To avoid potential bias induced by a lack of blinding, we will use an objective outcome (HbA1c) as the primary outcome and will ensure blinding of outcome assessors.

➢ The generalizability of the results generated from this trial will need to be assessed through multicenter trials.

## Background

Persistent glycemic control is one of the key treatment objectives in the management of type 1 diabetes mellitus (T1DM) to prevent patients from developing and experiencing progression of microvascular and macrovascular complications that lead to disability and premature death [1]. Although insulin injections are effective evidence-based strategies for treating T1DM [2], the management status of T1DM is far away from optimal. For example, according to the data from the T1D Exchange Clinic Network, the percentages of patients achieving the glycated hemoglobin (HbA1c) target set by the American Diabetes Association (ADA) ranged from 14% (18–25 years old) to 30% (26–49 years old) among T1DM patients in the USA in 2014 [3]. Poor T1DM control leads to a heavy disease burden, and it accounted for 964.3 years lived with disability (YLDs) per 1000 globally in 2017 [4]. The situation is even worse in China. According to limited data from hospital-based T1DM patients, there were 3.8 events of diabetic ketoacidosis per 100 patient years among T1DM patients who had a disease duration of more than 1 year [5].

To optimize the benefits of insulin, skills aimed at mastering the adjustment insulin doses are essential and emphasize the important role of diabetes self-management education (DSME) program. Over the past 20 years, DSME programs had been shown to be efficacious and cost-effective in improving patients’ knowledge, skills, and motivation, all of which had contributed to the improvement of biomedical, behavioral, and psychosocial outcomes. For T1DM patients, evidence had demonstrated that structured education programs (SEPs) were the most effective strategy among various DSME programs [6]. However, the implementation of SEP was not optimal due to the complexity of the educational materials and lack of resources (e.g., skill, time, and staff) [7]. Furthermore, even after the successful implementation of SEPs that helped patients improve blood glucose control and quality of life [8], the results from studies with longer follow-up durations showed that HbA1c rebounded 1–3 years after a SEP [9], suggesting that the traditional face-to-face group education model could not satisfy the need for lifelong blood glucose management in T1DM patients.

In the digital era, mobile health can be a possible solution for overcoming the drawbacks of the traditional education model. Evidence had demonstrated the therapeutic effects of smartphone apps for diabetes, with the majority of them being targeted at type 2 diabetes mellitus (T2DM) [10]. A recent systematic review identified seven randomized controlled trials (RCTs) that assessed the effectiveness of smartphone apps in controlling HbA1c in T1DM patients, with five apps specifically targeted at T1DM patients and two targeted at both T1DM and T2DM patients [11]. Pooled results indicated that compared to usual care, patients in the smartphone app group showed a significant reduction in HbA1c (pooled mean difference [MD] − 0.49%, 95% confidence interval [CI] − 0.94 to − 0.04%, *I*^2^ = 83.29%, 7 RCTs), with significant heterogeneity and low quality of evidence. Additionally, only one RCT [12], in which the app was not specifically targeted at T1DM patients, was conducted in China. This implies that an intervention based on internet and mobile medicine that is specifically targeted at the Chinese T1DM population in an evidence-based and culturally tailored manner is urgently needed for improving type 1 diabetes care in China.

A smartphone app with multiple functions (Yi tang yun qiao - The cloud bridge between clinicians and patients) aimed at facilitating a SEP and diabetes control for T1DM patients in the Chinese population was developed by our research team [13]. A needs assessment using mixed methods was used to collect quantitative and qualitative data from both diabetologists and T1DM patients regarding the functions that should be included in a diabetes app [13]. Then, a theoretical- and evidence-based app targeted at T1DM patients was successfully developed [13]. The incorporation of a digital SEP curriculum in the app is one of the main features of the app. To promote the efficiency of SEPs, artificial intelligence technology was used for automated, individualized SEP material delivery after assessment of a patient’s diabetes management skills. Details about the app development process were reported in a previous publication [13].

After considering the needs of both the patients and clinicians, we hypothesized that the implementation of the new SEP strategy would help improve diabetes control for T1DM patients. However, a well-designed RCT is needed to assess the effectiveness of the newly developed app compared to usual care. The present protocol is proposed to serve this purpose. The detailed hypotheses of the RCT are that T1DM patients using the app, as opposed to usual care, will have better diabetes control in terms of HbA1c level, time in range (TIR), quality of life, self-efficacy, and psychological conditions.

This paper presents the details of the design of and protocol for the first RCT assessing the effectiveness of a new SEP based on mobile technology that specifically targeted the T1DM population in China. This study is innovative in that it compares the effectiveness of smartphone apps between usual care and automated, individualized app education to explore whether individualized treatment, as advocated by the latest guidelines [14], will bring any additional benefit to T1DM patients. The ultimate goal is to provide an effective and convenient approach for T1DM control and reduce the related disease burden in China, where the T1DM control status is far from optimal. The protocol of the trial is reported by following the items recommended by the Standard Protocol Items: Recommendations for Interventional Trials (SPIRIT) 2013 statement [15]. The final results will be reported after the RCT in line with the Consolidated Standards of Reporting Trials (CONSORT) 2010 Statement for reporting parallel group randomized trials [16].

## Methods

### Overview of the study design

**Fig. 1:**
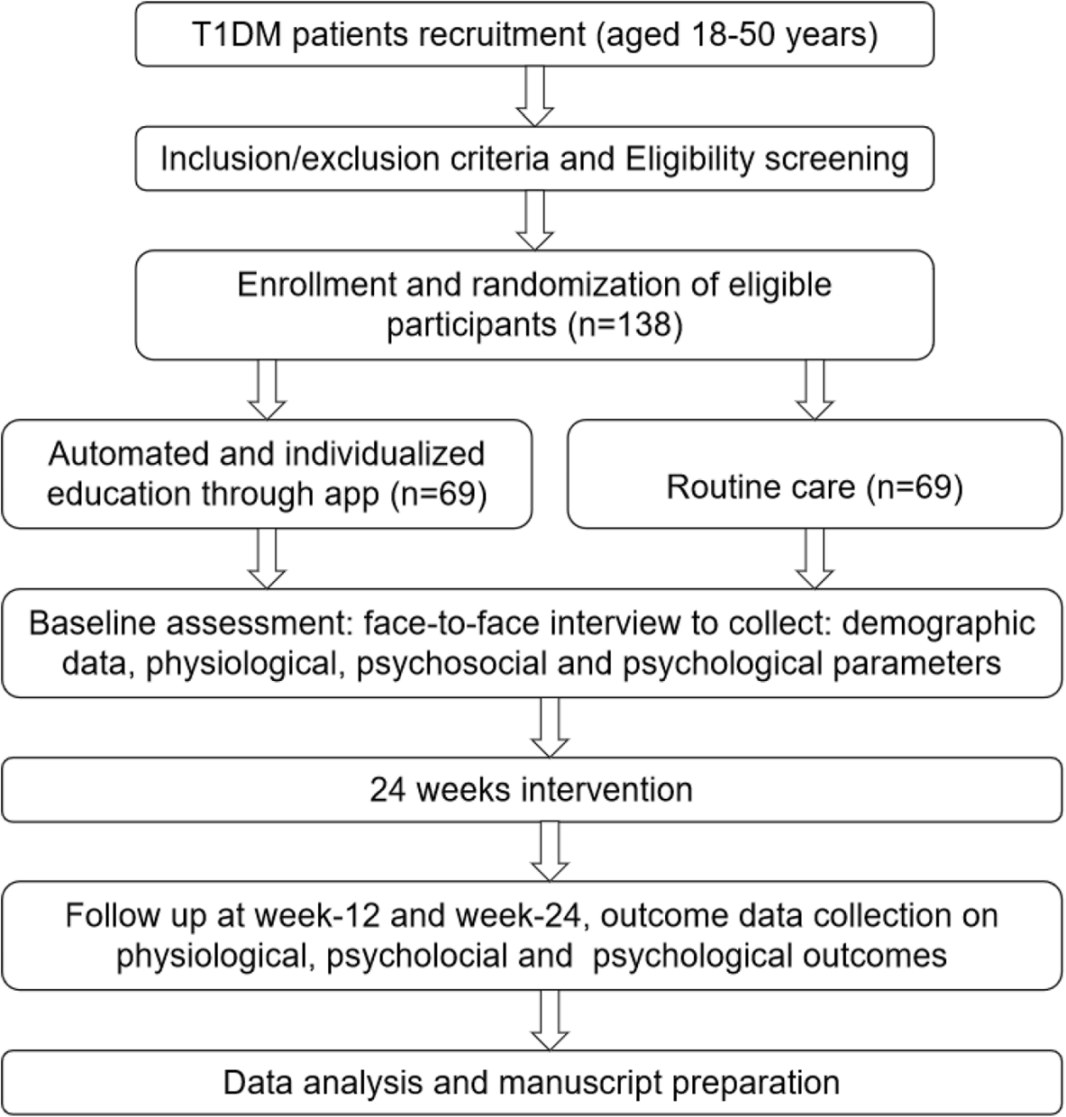
Flowchart of the trial

This single-blinded, 24-week, parallel-group randomized controlled trial with a 1:1 allocation ratio will be conducted in Changsha, China. Patients with T1DM will be randomly allocated into one of two groups: automated, individualized education through an app, and artificial intelligence or routine care. The intervention will last for 24 weeks. The primary outcome will be the change in HbA1c from baseline to week 24 (see flowchart in Fig. 1).

Outcome measurements will be collected at baseline, week 12, and week 24. The laboratory staff who test the HbA1c level; the outcome assessor who collects data on psychosocial, physiological, and anthropometric data; and the statisticians will be blinded to the treatment allocation. All the outcomes and their measurement sequences during the RCT are presented in Table 1.

**Table 1:** The sequence of trial measurements for the primary and secondary outcomes

Timepoint	Baseline	Week 12	Week 24
**Primary outcome**			
HbA1c	×	×	×
**Secondary outcome**			
Time in range (TIR)	×	×	×
Fasting blood glucose (FBG)	×	×	×
Systolic blood pressure	×	×	×
Diastolic blood pressure	×	×	×
Total cholesterol	×	×	×
High-density lipoprotein (HDL) cholesterol	×	×	×
Low-density lipoprotein (LDL) cholesterol	×	×	×
Triglycerides	×	×	×
Body mass index (BMI)	×	×	×
Adult diabetes quality of life (A-DQOL)	×	×	×
The type 1 diabetes self-management questionnaire	×	×	×
Summary of Diabetes Self-Care Activities Assessment (SDSCA)	×	×	×
Diabetes Empowerment Scale-Short Form (DES-SF)	×	×	×
Beck’s Depression Inventory (BDI)	×	×	×
State-Trait Anxiety Inventory (STAI)	×	×	×
Patients satisfaction	×	×	×
Adverse events^a^	×	×	×

^a^Adverse events will be collected every 4 weeks during the intervention period through a telephone interview

### Setting

The trial setting is a university-affiliated tertiary hospital, the Second Xiangya Hospital of Central South University, in Changsha, Hunan, China. The study received ethics approval from the ethics committee of the Second Xiangya Hospital, Central South University (2019 Ethics Approval [Department] No. 072), and was registered as NCT04016987.

### Participants

Participants who meet the diagnostic criteria for T1DM according to the 1999 World Health Organization (WHO) report [17] will be considered eligible. Detailed inclusion and exclusion criteria are listed in Table 2. There will be no special criteria for discontinuing the allocated interventions.

**Table 2:** Eligibility criteria

Inclusion criteria	Exclusion
1. Insulin dependence from disease onset	1. Age below 18 years or above 50 years
2. Disease duration longer than 6 months	2. Being pregnant
3. Age between 18 and 50 years old	3. Having a mental disorder
4. Positive testing for at least one of the three pancreas autoantibodies: glutamic acid decarboxylase autoantibody (GADA), insulinoma-associated-2 autoantibodies (IA-2A), zinc transporter 8 autoantibody (ZnT8A)	4. Having any other condition or disease that may hamper from compliance with the protocol or complication of the trial
5. HbA1c > 7.0%	5. Already using a smartphone app for managing diabetes
6. Being treated with multiple daily injections or insulin pump	6. Having chronic complications including diabetic retinopathy, diabetic nephropathy or diabetic foot, diabetic neuropathy
7. Owning a smartphone and are capable of sending a message through WeChat or an app	

### Sample size estimation

We propose to enroll 138 T1DM patients including withdrawals, 69 in the smartphone app group and 69 in the routine care group. The sample size estimation is based on the hypothesized changes in the primary outcome HbA1c. Details are presented below.

The primary analysis will compare the level of HbA1c between the smartphone app group and the routine care group. In a recent meta-analysis [11], data from seven RCTs that compared the difference in HbA1c between smartphone app and usual care groups were extracted, and the pooled mean difference was − 0.6%, which was used as the expected effect of this RCT. Using a two-sided hypothesis test at a 5% significance level, with an allocation ratio of 1:1, a total of 110 T1DM patients will be needed to detect a difference in HbA1c between the smartphone app group and routine care group of 0.6% with 80% power. The withdrawal rate among the RCTs included in the meta-analysis ranged from 0.0 to 26.4%, with a median of 8.4% [11]. We conservatively used a drop-out rate of 20%, yielding a total sample size of 138 (69 in the smartphone app group and 69 in the routine care group).

### Recruitment strategies

A combination of the following strategies will be employed for recruiting T1DM patients: (i) invitation letters will be sent to T1DM patients included in the T1DM cohort that has been established by our research team, (ii) flyers and posters will be posted within the hospital, and (iii) online advertisements will be published on WeChat—a social network platform—public accounts, and doctors and diabetologists will be asked to spread the information. WeChat has been chosen for its broad use among smartphone users in China [18]. Any interested respondents will be provided with the details about the trial and will be assessed for their eligibility by a diabetologist according to the eligibility criteria listed in Table 2 through a face-to-face consultation.

### Intervention assignments

#### Randomization and sequence generation

All participants who meet the inclusion criteria for participation and who sign the informed consent form will be randomized by an independent research member. Blocked randomization will be adopted to assign patients to the two groups at a 1:1 ratio with random block sizes [19]. The random sequence will be generated with SPSS 25 by a statistician [20].

#### Allocation concealment

Allocation concealment will be achieved through sequentially numbered, opaque sealed envelopes. The random sequence will be generated by a statistician who will not be involved in the patient enrollment and baseline assessment process. The envelope will only be opened for each patient after consent is obtained, eligibility is confirmed, and the baseline evaluation is finished.

#### Blinding

Due to the nature of the smartphone app used in this RCT, it is impossible to blind patients and clinicians. However, blinding of outcome assessors and data analysts will be ensured. Data entry will be accomplished by individuals external to the research team, and data analysis will be completed without referring to the allocation information. Patients and clinicians will be strongly discouraged from disclosing group assignment information to the outcome assessors. To overcome the lack of participant and clinician blinding, the objective outcome HbA1c has been chosen as the primary outcome. It is well recognized that a lack of blinding has a minimal impact on the assessment of objective outcomes [21].

### Intervention

The study interventions will last for 24 weeks. Patients in each of the two groups will be encouraged to continue their previous insulin treatment regimen, with either multiple daily injections or insulin pumps. All the interventions will run concurrently. Patients allocated to the smartphone app group—the automated, individualized education group—will attend a course on the use of the app provided by the research team. The course will be given to the two groups of patients separately to reduce possible contamination.

#### Automated and individualized education through an app and artificial intelligence

**Fig. 2:**
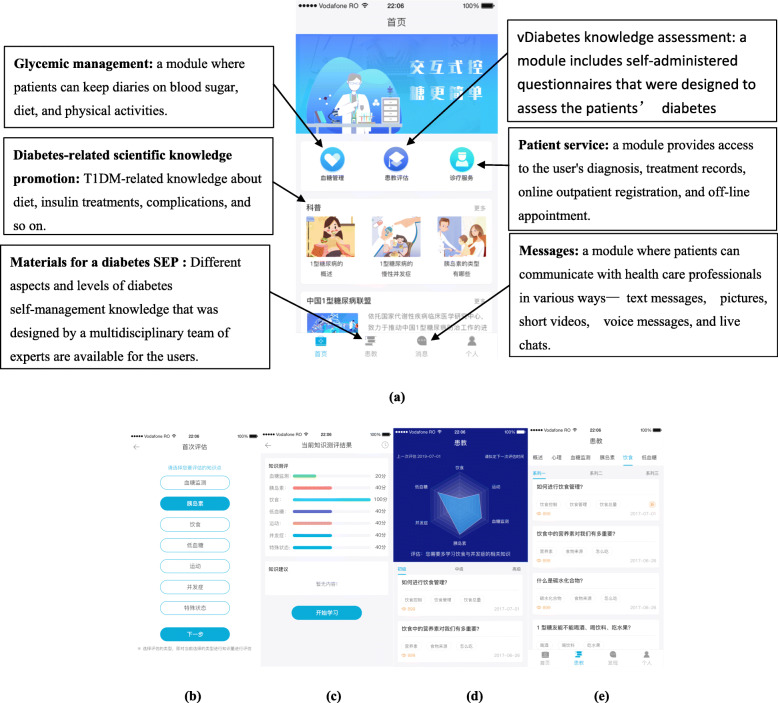
Homepage of the smartphone app (Yi Tang Yun Qiao). **a** The English translation and explanation of homepage. **b**–**d** The diabetes knowledge assessment module. **e** Different aspects and levels of SEP diabetes education materials. Keys: SEP, structured education program

Patients allocated to the individualized group will be given instructions to install the app, which includes the following modules: glycemic management, diabetes knowledge assessment, diabetes-related scientific knowledge promotion, patients’ service, materials for a diabetes SEP, and messages (Fig. 2).

**Fig. 3:**
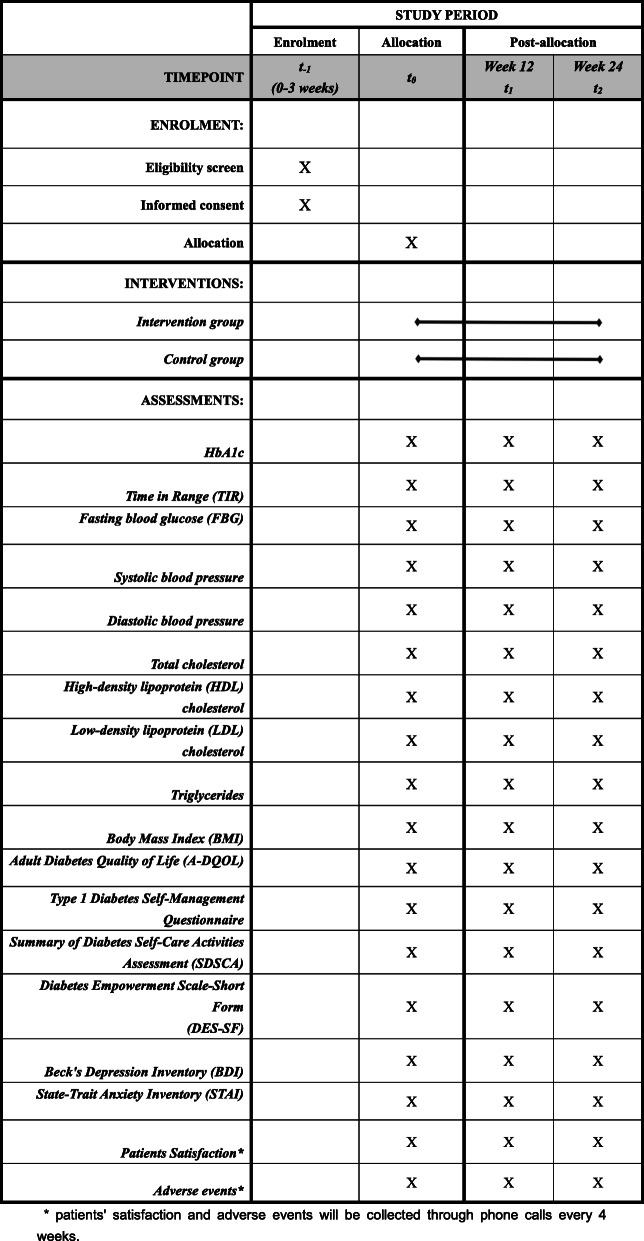
Full Standard Protocol Items: Recommendations for Interventional Trials (SPIRIT) figure

Through the glycemic management module, patients can keep diaries on blood glucose, diet, exercise, medication, height, and weight. To reduce the data entry burden, the results on blood glucose could be transferred from glucose meters to the app through Bluetooth or General Packet Radio Service. Daily step count data could be obtained from the step counter software in the smartphone, and diet information could be recorded by taking photos of the food through the built-in camera [13].

The diabetes knowledge assessment module (Fig. 2b–d) includes self-administered questionnaires that were designed to assess patients’ diabetes-related knowledge. The module serves to assess patients’ baseline diabetes-related knowledge. A popped-up box will be used as a reminder each time patients begin browsing the diabetes SEP in the automated, individualized education group.

The diabetes-related scientific knowledge promotion module provides general knowledge on T1DM, including an overview of T1DM, complications related to T1DM, insulin treatment, etc.

The patient’s service module provides access to electronic medical records including (i) the laboratory results (e.g., HbA1c test results) and (ii) diagnosis and treatment records (e.g., diabetes-related complications and their corresponding treatments). This module will keep both patients and clinicians well informed about a particular patient’s diabetes care. Online outpatient registration and off-line appointments with clinicians are also available for patients.

The diabetes SEP material (Fig. 2e) includes two parts. Part 1 serves the individualized education purpose, which has a push notification function that provides recommended education materials that meet the needs of the patient by considering his/her baseline diabetes-related knowledge. Part 2 provides a database that includes digital SEP material developed by our research team. Patients can browse or choose any content according to their interests. No push notification is included in part 2.

The message module facilitates patient-doctor communication through various means: typing words, sending pictures or short videos, sending voice messages, and live chatting. The patient and his/her doctor will establish a blood sugar target collaboratively. The doctor will review the patients’ diabetes diary once a week and provide feedback to patients: (i) give positive feedback if patients are keeping the target well, (ii) alert patients if they are off-target, and (iii) provide other tailored feedback by considering patients’ conditions. Furthermore, whenever a patient’s blood glucose level is dangerous (< 3.9 mmol/L or > 20.0 mmol/L), an alert will be automatically sent to the doctor, and an immediate phone call will be made to the patient by the doctor [13].

#### Routine care

Patients allocated to the routine care group will receive the education provided by health-care professionals in the outpatient department. They will not be asked to use any smartphone app during the trial. This trial will not require alteration in the use of any medication to both trial arms.

### Approaches to improve adherence to the intervention

Two strategies will be utilized to encourage the participants’ usage of and adherence to the recommendations of the app. First, adherence reminders will be provided by our health care professionals during every face-to-face visit at the outpatient department and telephone interviews every 4 weeks. Second, we will award the top three most frequent users every month with small gifts.

### Outcomes

Both subjective and objective outcomes will be included in the outcome measurements. Due to the nature of the study design, it is impossible to blind the patients and personnel in the trial. Therefore, we will choose HbA1c, a laboratory-tested index, to reduce potential bias induced by the lack of blinding of participants and personnel. We will also include patient-reported outcomes (e.g., quality of life) to provide a comprehensive assessment of the treatment effect. Detailed explanations of the outcome measurements are provided below.

#### Primary outcome

HbA1c has been chosen as the primary outcome for its ability to reflect patients’ average level of blood glucose over the past 2 to 3 months. It is the gold standard for evaluating the long-term effect of glycemic management and has been proven to be associated with the risk of developing various complications [22]. Peripheral blood will be collected during the hospital visit at the request of the patients’ doctor during each scheduled follow-up time point (baseline, week 12, and week 24). The HbA1c test will be performed by the Central Laboratory, which has been certificated by the International Federation of Clinical Chemistry and Laboratory Medicine (IFCC).

#### Secondary outcomes

Due to the complex nature of T1DM, it is important to include a wide spectrum of outcomes to measure the treatment effect. Therefore, physiological parameters, psychosocial outcomes, and psychological outcomes are included in the secondary outcomes.

Time in range (TIR) of 70–180 mg/dL (3.9–10 mmol/L) will be used as a secondary outcome to overcome the inability of HbA1c to provide information on patients’ hypoglycemia, glycemic variability, or daily patterns of glycemia [23]. TIR is a newly recognized indicator of glycemic management by both clinicians [23] and patients [24]. TIR data will be collected using a continuous glucose monitoring system (iPro2, Medtronic). Data on other physiological parameters including fasting blood glucose (FBG), levels of cholesterol and triglycerides, blood pressure, and body mass index (BMI) will be also collected as the secondary outcome (Table 1).

The valid and reliable diabetes quality of life (DQOL) scale [25] is a widely used 46-item tool for assessing the quality of life related to diabetes in terms of three aspects: diabetes satisfaction (15 items), impact (20 items), and worry (11 items). Each item is answered on a 5-point Likert scale, with a score of 1 representing “always affected,” “always worried,” or “never satisfied” and a score of 5 indicating “no impact,” “no worries,” or “always satisfied”. A higher total score reflects a better quality of life. A Chinese version of the DQOL scale has been translated [26] and validated [27] in the diabetic population from mainland China and will be adopted in this trial.

Diabetes self-care behaviors will be assessed with the Summary of Diabetes Self-Care Activities (SDSCA) measure, which contains six behavior-related scales: general diet, specific diet, glucose monitoring, physical activity, foot care, and smoking. Absolute weekly frequency or consistency of diabetes self-care activities are scored with a scale ranging from 0 to 7, with higher scores reflecting better performance in self-care behaviors. The internal consistency reliability and construct validity of the SDSCA was supported by its psychometric test based on an adult diabetes population [28]. A validated Chinese version of the SDSCA is available for this trial [29].

Patients’ diabetes-related psychosocial self-efficacy will be evaluated with the Diabetes Empowerment Scale-Short Form (DES-SF), which is a short form of the Diabetes Empowerment Scale developed based on the American population with type 1 or type 2 diabetes [30, 31]. A revised Chinese version is available for the mainland China population [32]. The Chinese version of the DES-SF includes 8 domains with 1 item for each, covering the need for change, making a plan, overcoming barriers, seeking support, maintaining optimism, supporting oneself, diabetes-related pressure relief, and making the appropriate choice according to individual conditions for self-diabetes care. Each item is answered on a 5-point Likert scale, with 1 indicating strongly disagree and 5 indicating strongly agree. The total score ranges from 8 to 40, with a higher total score reflecting better psychosocial self-efficacy.

Patient psychological status, including depression and anxiety, will be assessed with the Beck’s Depression Inventory (BDI) and the State-Trait Anxiety Inventory (STAI) respectively. The Chinese version of the BDI consists of 21 self-rated items [33]. Each item will be scored from 0 to 3, with a total score ranging from 0 to 63, and a higher score indicates more serious depression. The Chinese version of the STAI consists of two subscales to measure both state and trait anxiety states [34]. Each of the two anxiety states will be measured with a 20-item subscale, and each item will be scored from 1 to 4. The total score for both state and trait anxiety ranged from 20 to 80, with higher scores indicating more serious anxiety. Data on patients’ satisfaction will also be collected through verbal rating grading, which involves ordering a four series of adjectives from lightest to strongest satisfaction.

Patients’ engagement with the app will be measured in terms of communications with the clinician and the utilization of the smartphone app. Specifically, the number of messages with education materials sent to the patients, the number of and delay of in message responses, the number of video calls/phone calls with patients, the number of logs entered entries by patients, and the time spent in the diabetes SEP diabetes education module will be collected.

Safety-related outcomes, including hypoglycemic events, hospitalization, and emergency room visits, will be collected at each follow-up time point.

### Data management and statistical analysis

#### Data collection

Primary and secondary outcomes will be collected by qualified research members at three face-to-face follow-up time points: baseline, week 12, and week 24. Data input will be double-checked by two independent research members. Patients’ compliance data and the number of communications with patients through the app will be collected and stored in the backend database of the app. Safety-related outcomes will be collected at each follow-up time point, including telephone interviews.

#### Data management

All the hardcopies of the patient report forms (PRFs) will be retained in locked cabinets in numerical order. Access to the PRFs and trial data will be restricted. All collected data will be transformed into electronic forms weekly. All data will be entered electronically by individuals not involved in the trial within 1 week after the data collection. The entered data will be kept in a separate secure location as an electronic backup. Double data entry will be adopted to reduce manual mistakes. Two versions of the entered data will be compared, with disagreements resolved by referring to the original hardcopy PRF. A random subset of data will be selected for quality control purposes after the completion of data entry. Data from selected PRFs will be compared against the entered data. Further mechanisms will be used for data integrity, including referential data rules, range checks, valid value checks, and consistency checks. All amendments of data entered in the database will be documented as well. Interim auditing of the RCT will be conducted upon recruiting half of the proposed number of patients.

#### Data analysis

The primary data analysis will be conducted by using data collected at week 24 under the intention-to-treat principle by including all the randomized patients in the data analysis. Missing data will be filled in with a multiple imputation method under the “missing at random” assumption [35]. Any substantial difference in baseline characteristics will be adjusted with mixed-model regression analysis. Between-group differences will be assessed for the smartphone app group versus the routine care group. Relative risk reductions with 95% CIs will be calculated for dichotomous outcomes. The mean difference with standard deviation (SD) will be used to measure the treatment effect for continuous outcomes.

Secondary data analysis will use data collected at week 12 and week 24. All analyses will follow the same template as the primary data analysis by using a mixed model to adjust for imbalanced baseline characteristics. Linear regression will be used for continuous outcomes, and logistic regression will be used for binary outcomes. Model assumptions will be checked by regression diagnostics for all regression models. Predefined moderators including psychological and psychosocial mediators (e.g., depression, anxiety, self-efficacy) will be examined for treatment interactions. The Bonferroni method will be adopted to adjust the significance level under the circumstance of multiple testing. Furthermore, longitudinal analysis in which data collected at baseline, week 12, and week 24 will be jointly analyzed with appropriate mixed models. The time point will be included as a categorical fixed factor with the first-order autocorrelation of the errors and random intercepts. Monthly data collected on HbA1c during the intervention period will be analyzed with similar mixed models.

A sensitivity analysis will be conducted by using per-protocol data and excluding those patients who drop out of the RCT. The results from the sensitivity analysis might provide some pragmatic evidence from the real world regarding the effectiveness of the smartphone app for controlling T1DM. All statistical tests will be two-sided, with *p* < 0.05 considered to indicate statistical significance.

#### Data monitoring

The Data Monitoring Committee (DMC), the Ethics Committee and Trial Steering Committee (TSC) of Second Xiangya Hospital, the latter consisting of endocrinologists, diabetes nurses, nutrition scientists, registered dietitians, certified diabetes educators, and behavioral scientists, will be responsible for monitoring the trial conduct and patient safety and making recommendations for trial modifications or termination based on benefit-risk assessments. The principal investigator (XL) can then decide whether to make changes. The TSC will meet at least once a month to oversee the trial conduct and progress. The frequency of interim analyses will depend on the judgment of the DMC in consultation with the PI.

The DMC, the Ethics Committee, and the TSC are independent of the trial organizer. The chair is Dr. J.Z. Terms of reference for the DMC are available on request from the Clinical Drug Trial Institution at Second Xiangya Hospital.

### Protocol amendments

Any changes to the protocol will involve notification of the trial sponsor first and then the centers, and the clinical trial registry will be updated. A copy of the revised protocol will be added to the Investigator Site File by the PI. Any deviations from the protocol will be fully documented using a breach report form.

### Patient and public involvement

Patients with T1DM were involved in the development of education materials for this study. Patients who meet the inclusion criteria will be recruited and involved in the conduct of the trial. The feasibility of the app will be evaluated by all participants via monthly phone interviews. Study results will be disseminated to the participants on request.

## Discussion

A robust and well-designed RCT will be conducted to determine the effect of an automated, structured education intervention based on an app and artificial intelligence; it has been developed in an evidence-based and culturally tailored manner for the Chinese population for the management of T1DM. The detailed research plan of the RCT is presented in this protocol.

Although evidence had established the effect of smartphone apps in diabetes care, the majority of apps had been developed for T2DM patients and targeted at populations outside of China [10, 11]. According to an updated systematic review [11], only seven trials provided evidence on the effect of smartphone apps among T1DM patients. Pooled results (pooled MD in HbA1c − 0.49%, 95% CI − 0.94 to − 0.04%, *I*^2^ = 83.29%) showed a promising benefit for using smartphone apps to manage T1DM. However, the results from the individual trials were substantially inconsistent (MD of HbA1c varied from − 1.17% [36] to 0.41% [37]), which explained the high level of heterogeneity (*I*^2^ = 83.29%) in the meta-analysis. Furthermore, the results also indicated that the effect of smartphone apps in type 1 diabetes care was still controversial and had a low quality of the evidence [11].

Hence, whether smartphone apps will provide benefits to T1DM patients has not yet been conclusively determined. Very limited effective apps are available for T1DM patients from China. We developed a smartphone app by specifically considering the cultural background and lifestyle of the Chinese population to assist self-management and meet the need for lifelong diabetes care in T1DM patients. In this study, we seek to determine the effectiveness of the newly developed app in type 1 diabetes care compared to routine care.

Moreover, we also plan to explore whether tailoring health education materials by considering the specific knowledge gaps of each individual patient will provide some extra benefits in reducing HbA1c and improving other psychological and psychosocial outcomes. This will facilitate the development of future smartphone apps. As advocated by the latest guidelines, strategies for diabetes management should promote individualized targets and treatments [14]. By including multiple secondary outcomes, this RCT has the chance to explore the potential mechanisms of smartphone apps in improving diabetes care. This will contribute to appropriate outcome selections for future RCTs in diabetes care.

Thus, successful implementation of the proposed RCT will contribute to the evidence base of whether a newly developed smartphone app is superior to routine diabetes care in the Chinese T1DM population. If it is proven to be effective, this study will provide a strategy that satisfies the need for effective lifelong diabetes care to reduce the disease burden and related complications caused by T1DM. The results obtained from this trial are expected to provide important public health implications for T1DM patients in China.

## Trial status

Participants’ recruitment began in September 2020 and was estimated to complete by the end of December 2021. This protocol is version 1.

## Supplementary information

**Additional file 1.** SPIRIT 2013 Checklist: Recommended Items to Address in a Clinical Trial Protocol and Related Documents.

## Data Availability

Data from the trial will be available upon request. The results will be submitted to peer-reviewed journals for publication and disseminated at both national and international conferences. Informed consent forms and other related documentation are available from the corresponding author upon request.

## Abbreviations

ADA: American Diabetes Association
A-DQOL: Adult diabetes quality of life
BDI: Beck’s Depression Inventory
BMI: Body mass index
CI: Confidence interval
CONSORT: Consolidated Standards of Reporting Trials
DES-SF: Diabetes Empowerment Scale-Short Form
DSME: Diabetes self-management education
FBG: Fasting blood glucose
GADA: Glutamic acid decarboxylase autoantibody
HbA1c: Glycated hemoglobin
HDL: High-density lipoprotein
IA-2A: Insulinoma-associated-2 autoantibodies
IFCC: International Federation of Clinical Chemistry and laboratory medicine
LDL: Low-density lipoprotein
MD: Mean difference
PRFs: Patient report forms
RCTs: Randomized controlled trials
SD: Standard deviation
SDSCA: Summary of Diabetes Self-Care Activities Assessment
SEP: Structured education program
STAI: State-Trait Anxiety Inventory
SPIRIT: Standard Protocol Items: Recommendations for Interventional Trials
T1DM: Type 1 diabetes mellitus
T2DM: Type 2 diabetes mellitus
TIR: Time in range
WHO: World Health Organization
YLDs: Years lived with disability
ZnT8A: Zinc transporter 8 autoantibody
